# Effects of Prepartum L-Tryptophan Supplementation on the Postpartum Performance of Holstein Cows

**DOI:** 10.3390/ani14091278

**Published:** 2024-04-24

**Authors:** Xuening Liu, Songyang Yao, Yunjie Liu, Huigang Han, Weijia Wang, Qi Yi, Laiqing Yan, Pengyun Ji, Lu Zhang, Guoshi Liu

**Affiliations:** Beijing Key Laboratory for Animal Genetic Improvement, Key Laboratory of Animal Genetics and Breeding of the Ministry of Agricultural, State Key Laboratory of Animal Biotech Breeding, National Engineering Laboratory for Animal Breeding, College of Animal Science and Technology, China Agricultural University, Beijing 100193, China; liuxuening20@163.com (X.L.);

**Keywords:** L-tryptophan, melatonin, prepartum, antioxidant, postpartum pregnancy rate

## Abstract

**Simple Summary:**

The peripartum period is a challenging phase in the production of dairy cattle. Tryptophan is an essential amino acid in animals and has a variety of physiological functions. This study examined the effects of L-tryptophan supplemented to Holstein cows during the prepartum period on the postpartum performance. We found that L-tryptophan supplementation in the prepartum period significantly improved the reproductive, antioxidative and anti-inflammatory performance of cows compared to the controls. All these lead to the improved quality of colostrum and milk yield, indicating a healthy transition of the cows from their prepartum to postpartum status.

**Abstract:**

The negative energy balance occurring in the periparturient period of cows will impede their health and postpartum performance. To target this issue, L-tryptophan was supplied to the prepartum cows. The results showed that L-tryptophan supplementation significantly increased the serum melatonin level and was accompanied with increases in SOD activity, IL-10 and colostrum IgA levels as well as decreases in MDA and IL-6 levels compared to the control cows. The incidence of postpartum diseases was significantly lower and the pregnancy rate was significantly higher in cows fed L-tryptophan than in the control group. A striking observation was that prepartum L-tryptophan supplementation not only improved the milk production but also the quality compared to the control cows. In general, supplementation with L-tryptophan in the prepartum period can improve the postpartum reproduction and lactation performance of cows to some extent.

## 1. Introduction

It is well known that the nutritional requirements for pregnant cows, especially in the perinatal period, are dramatically increased due to the rapid development of the fetus and the initiation of colostrum secretion. The challenge is that during this period the cow’s dry matter intake is significantly reduced, resulting in relatively low nutrients and energy availability for pregnant cows [1,2]. The low nutrient intake will lead to a state of negative energy balance (NEB) in cows in the perinatal period, and in order to compensate for the NEB, the body will actively mobilize fat storage and other reserved resources for extra energy production. Excessive mobilization of body fat may cause other negative physiobiological alterations. These include but are not limited to (1) increasing the metabolic burden of the liver, to release acute-phase proteins and alter the function of organs [3]; (2) increasing the production of free radicals, which can cause oxidative stress in the related tissues and organs [4]; (3) reducing the immune function and lowering the cow’s ability to resist invasion by pathogens, therefore resulting in an increase in disease susceptibility [5]; and (4) increasing other metabolic diseases such as abomasum displacement, mastitis and postpartum paralysis [6]. As a result, proper periparturient management and additional nutrient supplementation for pregnant cows are critical not only for the health of the mother but also for their offspring.

L-tryptophan is an essential amino acid for mammals and is not only involved in protein synthesis but is also a precursor of many important substances that are required for normal growth. These include 5-hydroxytryptophan, melatonin, niacin and NAD+. The metabolic active domain of L-tryptophan is its indole ring [7]. It is well documented that L-tryptophan has a wide range of physiological roles including being a substrate of protein synthesis [8], improving growth performance [9] and feed utilization [10], reducing stress [11] and liver fat content [12], and upregulating immunity [13]. Not only L-tryptophan per se, but its metabolites also participate in various fundamental biological processes including regulation of cell growth and division as well as antioxidant function. Thus, its deficiency will lead to a spectrum of adverse consequences for mammals. One of the most important derivatives of L-tryptophan is melatonin. In mammals, melatonin can only be synthesized from this essential amino acid with several enzymatic processes [14]. Melatonin has various physiological functions, including regulation of circadian and endocrine rhythms, promotion of animal reproduction, and functioning as an anti-inflammatory, antioxidative, anti-anxiety and analgesic molecule [15,16,17]. As such an important substance, the importance of L-tryptophan has yet to be investigated in peripartum cows. Considering the various physiological functions of L-tryptophan mentioned above and the special physiological conditions of periparturient cows, we selected this essential amino acid as an additional nutrient to feed perinatal cows during the last four weeks of their parturition. In this study, L-tryptophan was supplemented into the basal diet of periparturient Holstein cows at the stage of the prepartum period to investigate its potential effects on the health, postpartum lactation and reproductive performance of the cows.

## 2. Materials and Methods

### 2.1. Chemical Agents

L-tryptophan was purchased from Xian Musen Bioengineering Co., Ltd. (Xi’an, China).

### 2.2. Experimental Design

The animal study was performed in accordance with the provisions of the China Agricultural University Laboratory Animal Welfare and Animal Experimental Ethical Inspection Committee. The approved protocol number was AW01602202-1-5.

The animal study was conducted on a commercial farm (the cattle farm of Nankou) located in Beijing, China. The one hundred and thirty-five multiparous Holstein cows with disease-free and similar body conditions (2–3 parities, around 700 kg) were selected. The cows were divided equally into three groups, one control group and two experimental groups. The two experimental groups were given L-tryptophan either 50 g/d or 100 g/d with TMR (Table 1) at 6:00 a.m. daily. Treatments were offered from d −26 ± 2 d to parturition.

### 2.3. Serum Indicator Testing

Blood samples were obtained by venipuncture of the coccygeal vessels at −26 d, −21 d, −14 d, −7 d, 0 d, 7 d, 14 d and 21 d (at 1500 h), with 10 mL of blood per cow per collection. Serum was obtained after centrifugation at 1000× *g* for 10 min, and then kept at −20 °C until analysis. The serum samples were tested with the described method. Glucose, triglyceride and total cholesterol concentrations were measured using the ZY-1280 Automatic biochemical analyzer (Shanghai Kehua Bio-Engineering Co., Ltd., Shanghai, China). Malondialdehyde concentration, superoxide dismutase activity and total antioxidant capacity were measured using the L-3180 semi-automatic biochemical analyzer (Shanghai Kehua Bio-Engineering Co., Ltd., Shanghai, China). Non-esterified fatty acid, β-hydroxybutyric acid, very-low-density lipoprotein, cortisol, interleukin-6, interleukin-10, estradiol, progesterone, luteinizing hormone and follicle stimulating hormone in serum were determined by the enzyme-linked immunosorbent assay (ELISA), following the manufacturer’s instructions. Simply, the antibody binds to the enzyme complex and the content is then detected by color development. The ELISA kits were purchased from BeijipgJinHaiKeYu Biological Technology Development Co., Ltd. (Beijing, China).

### 2.4. Milk Indicator Testing

Milk samples were collected from four quarters at 1 d, 4 d, 7 d, 14 d and 21 d (at 1400 h), with 100 mL of milk into two tubes per cow per collection. We added a preservative to one of the tubes of milk and stored it at 4 °C for dairy herd improvement (DHI) determination, and the other tube of milk was stored at −20 °C until analysis. Colostrum IgG, IgM and IgA levels were measured using the ZY-1280 automatic biochemical analyzer (Shanghai Kehua Bio-Engineering Co., Ltd., Shanghai, China). Dairy herd improvement (DHI) data determination was performed by the National Milk Product Standard Sanction Laboratory located at the Beijing Animal Husbandry Station using a DHI measuring instrument (MilkoscanFT1, Serial No.91755049, Part No.60027086, made in Denmark).

### 2.5. Melatonin and Tryptophan Assay

Serum and milk samples were separately mixed with methanol in a 1:4 portion (1 mL:4 mL) and then oscillated in a vortex. After centrifugation (9300× *g* for 10 min), the supernatant was collected and filtered with a microporous membrane (0.22 μm) for use. Determination of melatonin and tryptophan levels in serum and milk was carried out by liquid chromatography tandem mass spectrometry (LC-MS/MS) in the central laboratory of the Beijing Institute of Animal Science, Chinese Academy of Agricultural Sciences (Beijing, China) using a high-performance liquid mass spectrometer (Agilent1290-G6470, Santa Clara, CA, USA).

### 2.6. Statistical Analysis

The data were presented as the mean ± SEM. One-way and two-way analyses of variance (ANOVA) were performed followed by Duncan’s multiple test using GraphPad Prism 8 software (La Jolla, CA, USA). The colostrum immunoglobulin data were analyzed by one-way ANOVA. The physiological and biochemical indicators and inflammatory factor data were analyzed by two-way ANOVA. Disease incidence rate and pregnancy rate were analyzed using chi-square test. The *p*-values < 0.05 were considered statistically significant.

## 3. Results

### 3.1. The Effects of Prepartum L-Tryptophan Supplementation on the Levels of Tryptophan and Melatonin in the Serum and Milk of Cows

**Figure 1:**
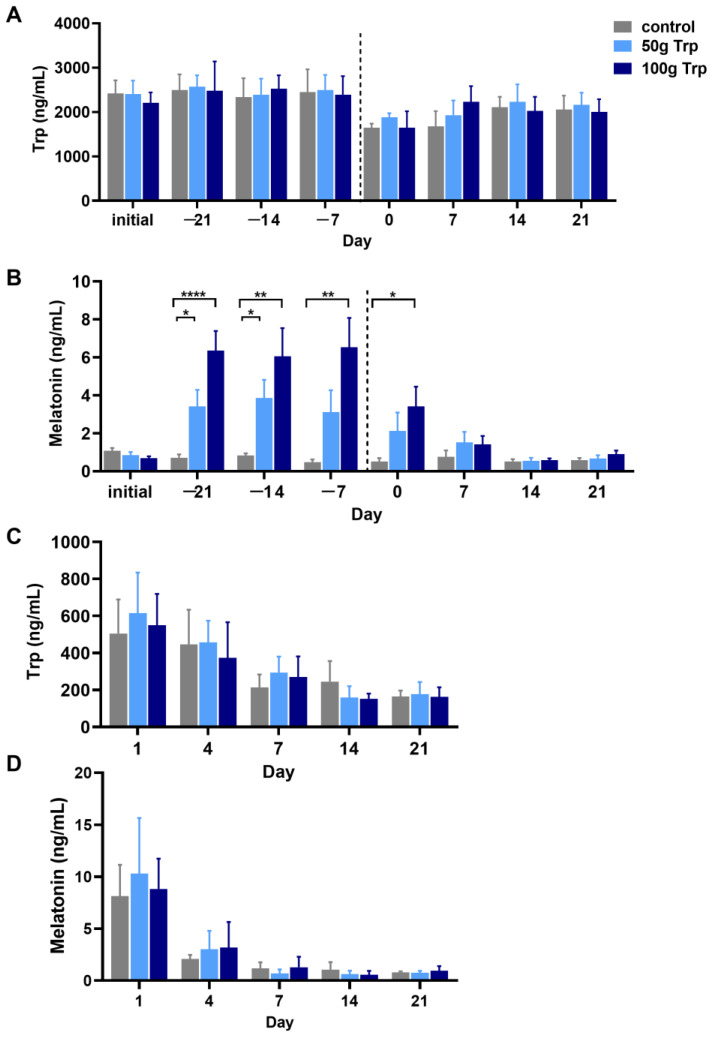
The effects of L-tryptophan supplementation on the levels of tryptophan and melatonin in serum and milk of the cows. (**A**) serum tryptophan content; (**B**) serum melatonin content; (**C**) milk tryptophan content; (**D**) milk melatonin content. “Initial” indicates sampling before cows were fed; −21, −14 and −7 indicate the number of days until the cows gave birth; 1, 4, 7, 14 and 21 indicate the number of days after the cows gave birth. The dotted line, day 0, represents the time when the cow calved and also when L-tryptophan supplementation stopped. All data are expressed by mean ± standard error. * *p* < 0.05, ** *p* < 0.01, **** *p* < 0.0001.

The results showed that L-tryptophan supplementation in the prepartum period of the cows had no significant effect on serum (Figure 1A) and milk (Figure 1C) levels of tryptophan as well as the melatonin level in milk (Figure 1D) compared to the control group (*p* > 0.05); however, the serum melatonin level was significantly higher in both 50 and 100 g L-tryptophan-treated groups than that in the control group at −21 d, −14 d, −7 d and 0 d (*p* < 0.05) (Figure 1B).

### 3.2. The Effects of Prepartum L-Tryptophan Supplementation on Glucose and Lipid Metabolism in Cows

**Figure 2:**
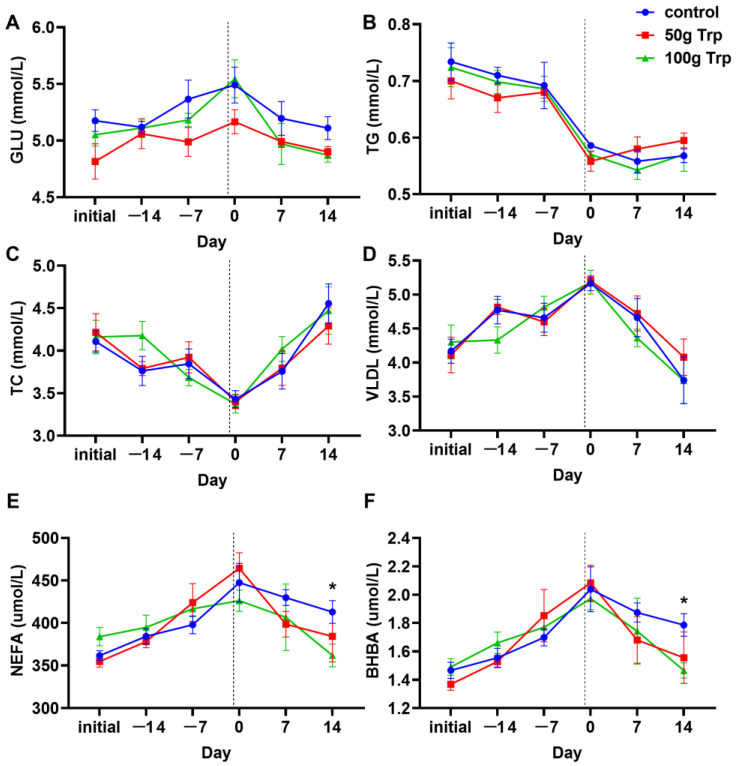
Effects of L-tryptophan supplementation on serum glucose and lipid metabolism in cows. (**A**) serum glucose (GLU) level; (**B**) serum triglyceride (TG) level; (**C**) serum total cholesterol (TC) level; (**D**) serum very-low-density lipoprotein (VLDL) level; (**E**) serum non-esterified fatty acid (NEFA) level; (**F**) serum β-hydroxybutyric acid (BHBA) level. “Initial” indicates sampling before cows were fed; −14 and −7 indicate the number of days until the cows gave birth; 7 and 14 indicate the number of days after the cows gave birth. The dotted line, day 0, represents the time when the cow calved and also when L-tryptophan supplementation stopped. All data are expressed by mean ± standard error. * *p* < 0.05.

The results showed that no significant differences in serum glucose (GLU) (Figure 2A), triglyceride levels (TG) (Figure 2B), total cholesterol (TC) (Figure 2C) and very-low-density lipoprotein (VLDL) (Figure 2D) were observed among the groups (*p* > 0.05). However, serum non-esterified fatty acid (NEFA) (Figure 2E) and β-hydroxybutyric acid (BHBA) (Figure 2F) levels in the 100 g L-tryptophan-treated group were significantly lower than that in the control group on day 14 after parturition (*p* < 0.05).

### 3.3. The Effects of Prepartum L-Tryptophan Supplementation on Immune and Antioxidant Performance in Cows

**Figure 3:**
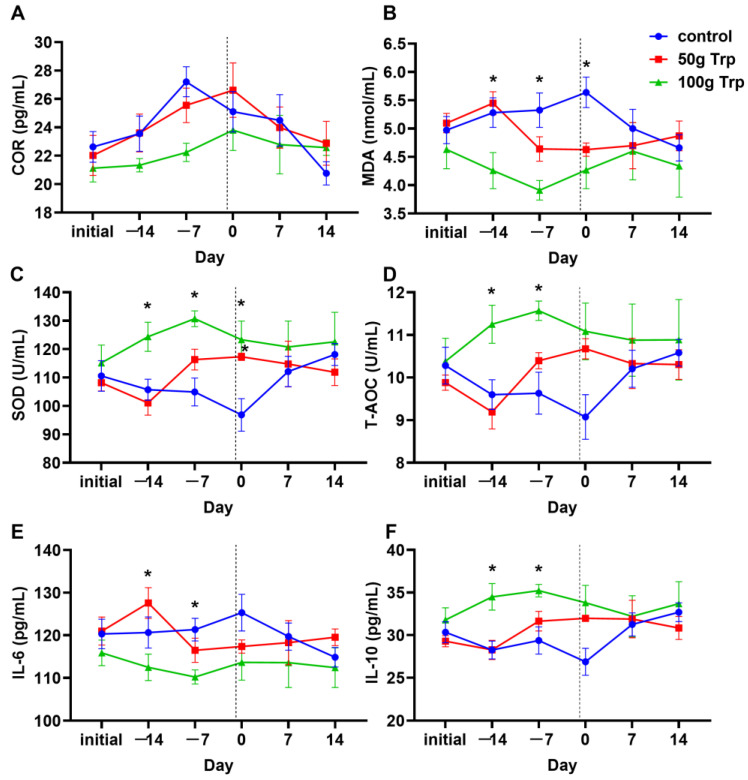
Effects of L-tryptophan supplementation on serum antioxidant and immune-related indexes of cows. (**A**) serum cortisol (COR) level; (**B**) serum malondialdehyde (MDA) level; (**C**) serum superoxide dismutase (SOD) level; (**D**) serum total antioxidant capacity (T-AOC) level; (**E**) serum interleukin-6 (IL-6) level; (**F**) serum interleukin-10 (IL-10) level. “Initial” indicates sampling before cows were fed; −14 and −7 indicate the number of days until the cows gave birth; 7 and 14 indicate the number of days after the cows gave birth. The dotted line, day 0, represents the time when the cow calved and also when L-tryptophan supplementation stopped. All data are expressed by mean ± standard error. * *p* < 0.05.

L-tryptophan supplementation in the prepartum period had no significant effect on serum cortisol (COR) levels among the groups (*p* > 0.05) (Figure 3A). The serum MDA content of the 100 g L-tryptophan-treated group was significantly lower than that of the 50 g L-tryptophan-treated group at −14 d, and was significantly lower than that of the control group at −7 d and 0 d (*p* < 0.05) (Figure 3B). Superoxide dismutase (SOD) activity was significantly higher in 100 g L-tryptophan-treated group than that of the control group at −14 d, −7 d and 0 d, and this increase in the 50 g group was only observed at 0 d (*p* < 0.05) (Figure 3C). Total antioxidant capacity (T-AOC) (Figure 3D) and interleukin-10 (IL-10) level (Figure 3F) of the 100 g L-tryptophan-treated group were significantly higher than those of the control group at −14 d and −7 d. The IL-6 level in 100 g L-tryptophan-treated group was significantly lower than that of the 50 g L-tryptophan-treated group at −14 d, and was significantly lower than that of the control group at −7 d (*p* < 0.05) (Figure 3E).

### 3.4. The Effects of Prepartum L-Tryptophan Supplementation on Postpartum Reproductive Performance in Cows

#### 3.4.1. Reproductive Hormones

**Figure 4:**
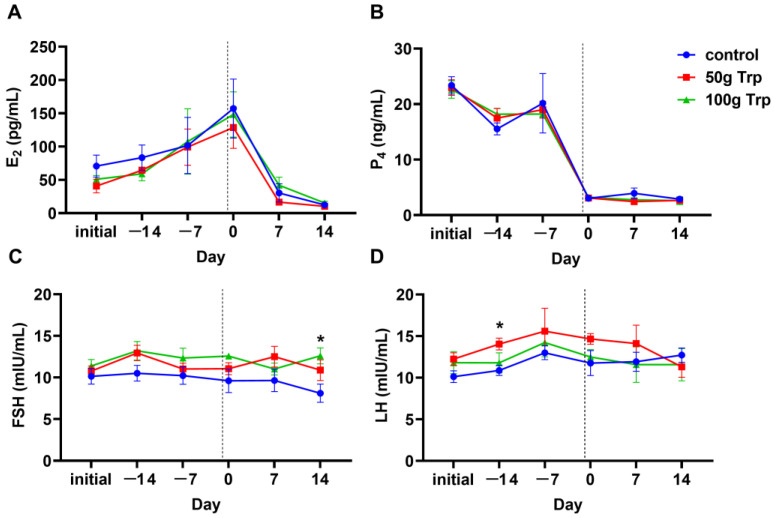
The effects of prepartum L-tryptophan supplementation on serum reproductive hormones of cows. (**A**) serum E2 level; (**B**) serum P4 level; (**C**) serum FSH level; (**D**) serum LH level. “Initial” indicates sampling before cows were fed; −14 and −7 indicate the number of days until the cows gave birth; 7 and 14 indicate the number of days after the cows gave birth. The dotted line, day 0, represents the time when the cow calved and also when L-tryptophan supplementation stopped. All data are expressed by mean ± standard error. * *p* < 0.05.

The results revealed that the trends in serum reproductive hormones in all groups were relatively consistent and in line with physiological patterns. L-tryptophan supplementation in the prepartum period had no significant effect on serum estradiol (Figure 4A) and progesterone levels (Figure 4B) compared to the control group (*p* > 0.05). The serum FSH levels were significantly higher in the 100 g L-tryptophan-treated group than that in the control group at 14 d (*p* < 0.05) (Figure 4C), while serum LH levels were significantly higher in the 50 g L-tryptophan-treated group than that in the control group at −14 d (*p* < 0.05) (Figure 4D).

#### 3.4.2. Postpartum Diseases in Cows

The effects of prepartum L-tryptophan supplementation on postpartum disease are listed in Table 2. The group receiving 50 g L-tryptophan showed no differences compared to the control group (*p* = 0.07). The incidence in the group receiving 100 g prepartum L-tryptophan was 20.00%, which was significantly lower than that in the control group (*p* < 0.05). Among the postpartum diseases, metritis was the most common disease in cows.

#### 3.4.3. Postpartum Pregnancy Rate in Cows

The effects of prepartum L-tryptophan supplementation on the postpartum pregnancy rate are listed in Table 3. The first postpartum breeding pregnancy rate and the two-time cumulative pregnancy rate in the 100 g prepartum-L-tryptophan-treated group were the highest, which were 22.22% (10/45) and 44.44% (20/45), respectively. The two-time cumulative pregnancy rate in the 100 g prepartum-L-tryptophan-treated group was significantly higher than that in the control group (*p* < 0.05).

### 3.5. The Effects of Prepartum L-Tryptophan Supplementation on Postpartum Colostrum Immunoglobulin Levels

**Figure 5:**
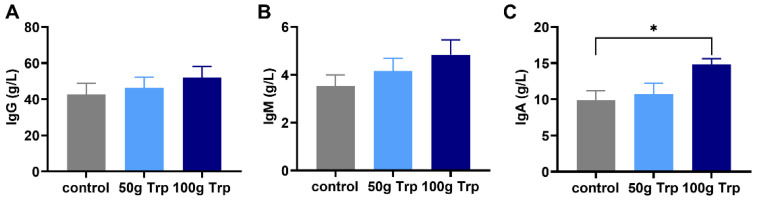
The effects of prepartum L-tryptophan supplementation on immunoglobulins in colostrum. (**A**) colostrum IgG content; (**B**) colostrum IgM content; (**C**) colostrum IgA content. All data are expressed by mean ± standard error. * *p* < 0.05.

The contents of IgG and IgM in the colostrum of dairy cows treated with L-tryptophan showed no significant differences compared to the control group (*p* > 0.05) (Figure 5A,B); however, the IgA content in the 100 g prepartum-L-tryptophan-treated group was significantly higher than that in the control group (*p* < 0.05) (Figure 5C).

### 3.6. The Effects of Prepartum L-Tryptophan Supplementation on Postpartum Daily Milk Yield and Compositions

**Figure 6:**
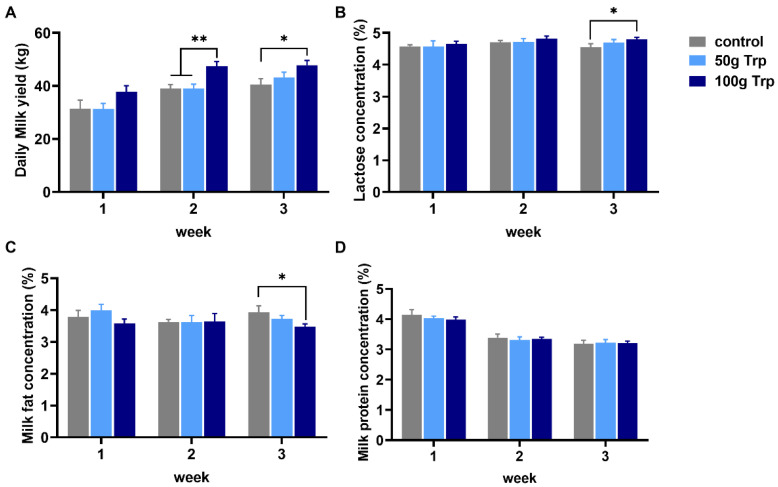
Effects of prepartum L-tryptophan supplementation on daily milk yield and milk compositions. (**A**) daily milk yield; (**B**) milk lactose concentration; (**C**) milk fat concentration; (**D**) milk protein concentration. All data are expressed as mean ± standard error. * *p* < 0.05, ** *p* < 0.01.

L-tryptophan supplementation in the prepartum period had a positive effect on milk yield. The milk yield in the 100 g prepartum-L-tryptophan-treated group was significantly higher than that in the other groups at postpartum weeks 2 and 3 (*p* < 0.05) (Figure 6A). The milk lactose concentration was significantly higher and milk fat concentration was significantly lower in the 100 g prepartum-L-tryptophan-treated group than those in the control group at postpartum week 3 (*p* < 0.05) (Figure 6B,C). No significant differences were observed in milk protein concentration among the groups (*p* > 0.05) (Figure 6D).

## 4. Discussion

The status of the perinatal period of the cows directly affects their postpartum health and their subsequent lactation cycle, which decides the milk yield. Serum levels of NEFA, BHBA, GLU, TG, TC and VLDL are important indicators of glucose and lipid metabolism in dairy cows. The results showed that L-tryptophan supplementation had no significant effects on the serum levels of GLU, TG, TC and VLDL levels compared to the control group. In addition, the serum levels of NEFA and BHBA were only significantly reduced in the 100 g L-tryptophan-treated group at 14 d, indicating that L-tryptophan supplementation did not have a significant effect on the glucose and lipid metabolism of dairy cows, or that tryptophan did not play a key determinant role. This may be related to the fact that tryptophan is not a key substance in glucose and lipid metabolism, as most of the current research on tryptophan focuses on its immunomodulation, stress reduction and other aspects [18,19].

MDA is the end product of lipid peroxidation and its content reflects the degree of free radical attack. The concentration of SOD and T-AOC activity are commonly used to evaluate the antioxidant status in animals. In the present study, L-tryptophan supplementation significantly reduced the concentration of MDA at −14 d, −7 d and 0 d, and increased the activity of SOD and T-AOC at −14 d and −7 d compared to the control group. IL-10 is an anti-inflammatory cytokine with an important role in the regulation of immunity and various inflammatory diseases [20]. We also observed that L-tryptophan supplementation significantly reduced the serum IL-6 and increased serum IL-10 levels at −14 d and −7 d compared to the control group. The results are consistent with previous reports in ducklings [21] and rats [22], suggesting the antioxidant and anti-inflammatory capacity of L-tryptophan. Melatonin is a potent antioxidant and immunoregulatory molecule, and it provides protective effects against oxidative stress and inflammation in a variety of ways [23,24,25]. In a previous study, we reported that the subcutaneous injection of melatonin significantly decreased the concentration of cortisol, and increased the content of IgM and IgG, and the number of white blood cells, neutrophils and lymphocytes in the blood of dairy cows, indicating that MT treatment can improve immune activity in cows [26]. We found that supplementation with L-tryptophan resulted in significantly higher serum levels of its derivative melatonin, which is consistent with several previous studies [27,28,29]. It is speculated that high levels of melatonin in the serum caused by L-tryptophan supplementation play a beneficial role in the improvement of the antioxidant and anti-inflammatory capacities of dairy cows.

In the current study, serum P4 levels in all three groups of cows remained high before parturition, declined rapidly to low levels on the day of parturition and remained low after parturition, while E2 levels peaked on the day of parturition and then declined to low levels. The hormonal trends in dairy cows were consistent with natural physiological patterns, showing that supplementation with L-tryptophan did not adversely affect the process of parturition. The levels of postpartum reproductive hormones can reflect the recovery of ovaries, and FSH can stimulate ovarian growth [30]. We found that at 14 days postpartum, the serum FSH content of cows in the group fed 100 g L-tryptophan was significantly higher than that of the control group, which means that the ovaries of cows were more active. A study reported that the time of first postpartum ovulation in dairy cows was (15.2 ± 0.8) d. However, the first estrus was mainly quiet estrus, which was related to the ratio of FSH to LH [31]. Importantly, the number of retained fetal membranes and metritis in the L-tryptophan-treated group was lower than that in the control group, and the number of healthy cattle was higher than that in the control group. The above results may explain this phenomenon, which helps cows to resist external pathogens and accelerates the recovery of the uterus and other organs after parturition, ultimately resulting in a lower probability of disease in cows. Low conception rates have been a challenge in dairy farming. The postpartum pregnancy rate of cows fed 100 g L-tryptophan was significantly higher than that of the controls, which may be related to the lower incidence of diseases and better uterine rejuvenation in the experimental group. Increased pregnancy rates contribute to shorter calving intervals for cows and higher economic benefits for the farm.

Immunoglobulins are the most important immunologically active substances in colostrum and regular milk, with IgA, IgG and IgM being the most studied [32]. In the current study, it was observed that the colostrum IgA level in the L-tryptophan-treated group was significantly higher than that in the control group. IgA has the ability to agglutinate antigens, neutralize viral and bacterial toxins, and prevent the adhesion of intestinal pathogens to the epithelial cells of the intestinal mucosa [33]. High immunoglobulin levels in colostrum will have a positive effect on improving the immunity and disease resistance of calves [34]. However, no significant differences were observed in colostrum IgG and IgM levels, which may be related to the different ways of regulating the secretion of IgA, IgG and IgM. A study in broilers concluded that increased serum IgG and IgM levels were associated with Toll-like receptor pathway protein expression [35].

Milk yield is affected by a number of factors, including breed, lactation stage, parity, illumination, disease, feed composition, nutrition and so on. The amount and composition of amino acids obtained from cow feed affects milk production and milk composition. Adding rumen-protected methionine to a diet based on corn, alfalfa silage and soybean cake increased milk production by 6% [36]. One of the important observations was that the prenatal L-tryptophan supplementation increased the milk yield. Kollmann et al. [37] reported that rumen bypass of L-tryptophan at 500 g/d-head (effective L-tryptophan 125 g/d-head), fed to lactating Swiss brown cows, significantly increased nighttime milk production. Another study on Holstein cows had similar results; they found that rumen-protected tryptophan supplementation improved milk yield [29]. A study found that tryptophan supplementation at 0.12% increased sows’ milk yield [9]. Paulicks et al. [38] found that with the increase in dietary tryptophan levels, the milk production of lactating female pigs showed a trend of first increasing and then decreasing. The increased milk production after parturition indicates a better condition of the cows with prenatal L-tryptophan supplementation. After calving, the output of nutrients with milk exceeds the input by voluntary feed intake, which can lead to a decline in milk production and quality [39,40]. This risk can be reduced by prenatal L-tryptophan supplementation since this significantly increased milk lactose and reduced the milk fat compared to the control group, suggesting the balanced energy metabolism and nutritional condition.

## 5. Conclusions

Based on our results, we conclude that L-tryptophan supplementation in the prepartum period had a positive effect on the postpartum lactation and reproductive performance of dairy cows. Although the dietary addition of L-tryptophan did not affect glucose and lipid metabolism, it did increase serum melatonin and IL-10 concentrations, SOD activity and T-AOC, and decreased MDA and IL-6 concentrations compared to the control cows. These results suggested that the antioxidant and anti-inflammatory capacities of the cows was enhanced by L-tryptophan supplementation to some degree. This view was supported by the lower disease incidence in cows in the L-tryptophan-treated group. These may be important factors in improving the quality of colostrum and milk yield, and the exact underlying mechanisms are currently unavailable and need further exploration. Surprisingly, supplementation with L-tryptophan improved conception rates in cows, and fewer inseminations means lower farming costs. The results are promising and may suggest the benefits of periparturient tryptophan supplementation in dairy cows.

## Figures and Tables

**Table 1:** Ingredients and chemical composition of the basal diet.

Item	%
Ingredient, % of feed	
Steam-flaked corn	3.50
Sprayed corn husk	2.20
Low-fat DDGS	2.20
Cereal grass	4.40
Domestic oats	13.20
Corn silage	57.00
Concentrate supplement ^1^	17.50
Nutrient composition, % of DM	
CP	15.00
NDF	44.00
NFC	34.70
EE	2.60
Trp (%mp) ^2^	1.46
Met (%mp)	2.16
Lys (%mp)	6.78

^1^ Concentrate supplement contained ground corn, soybean meal, soybean hulls, vitamin, Ca, P, Mg, Na, Cl, K, S, Mn, Zn, Cu, Se, I and Co as the main ingredients. ^2^ The meaning of %mp is amino acids as a percentage of metabolizable protein.

**Table 2:** Postpartum diseases in cows.

	Total Number	Healthy Number	Number of Cases	Disease Incidence (%)	Incidence of Retained Fetal Membranes	Incidence of Metritis	Incidence of Mastitis	Incidence of Lameness	Incidence of Postpartum Paralysis
Control	45	27	18	40.00 ^b^	3	7	5	1	2
50 g Trp	45	35	10	22.22 ^ab^	2	3	4	1	0
100 g Trp	45	36	9	20.00 ^a^	2	5	1	0	1

Note: Different lowercase letters in the same column indicate significant differences (*p* < 0.05) and the same letter indicates insignificant difference (*p* > 0.05).

**Table 3:** Postpartum pregnancy rate in cows.

	Total Number	Number of Pregnancies at First Breeding	Pregnancy Rate at First Breeding (%)	Number of Pregnancies at Two-Time Breeding	Cumulative Pregnancy Rate from Two-Time Breeding (%)
Control	45	5	11.11 ^a^	11	24.44 ^b^
50 g Trp	45	5	11.11 ^a^	16	35.56 ^ab^
100 g Trp	45	10	22.22 ^a^	20	44.44 ^a^

Note: Different lowercase letters in the same column indicate significant differences (*p* < 0.05) and the same letter indicates insignificant difference (*p* > 0.05).

## Data Availability

The data presented in this study are available on request from the corresponding author.
